# The informal healthcare providers and universal health coverage in low and middle-income countries

**DOI:** 10.1186/s12992-022-00839-z

**Published:** 2022-04-27

**Authors:** Emmanuel Kumah

**Affiliations:** Department of Health Administration and Education, Faculty of Science Education, University of Education, Winneba, Ghana

**Keywords:** Healthcare providers, Informal providers, Low-and-middle-income countries, Private sector, Universal health coverage

## Abstract

The World Health Organization has indicated that achieving universal health coverage (UHC) through public sector service delivery alone would not be possible. This calls for corporation, collaboration and partnership between the public and the private sector actors. Informal providers represent a significant portion of the healthcare delivery systems in low-and-middle-income countries (LMCs). However, the presence of this group of private sector actors in national health systems presents both challenges and opportunities. Considering the limited resources in LMCs, ignoring the role of the informal sector in national health systems is not an option. This paper aims to discuss the role of informal health care providers in achieving universal health coverage in low-and-middle-income countries.

## Background

The agenda 2030, comprising 17 Sustainable Development Goals (SDGs), provides a blueprint for global development [1]. The World Health Organization (WHO) has, however, indicated that although its member states have adopted the SDG agenda, achieving the health-related objectives through public sector service delivery alone would not be possible [2]. Thus, both the public and the private sector actors would need to corporate, collaborate and partner to meet the targets for SDG3, including the attainment of universal health coverage (UHC). According to WHO, UHC means that “all people are able to receive needed health services of sufficient quality to be effective, without fear that the use of those services will expose them to financial hardship” [1]. The concept comprises three key set of objectives: equity in access to health services, quality, and financial protection. It is based on the 1978 Alma-Ata declaration of Health for All and on the WHO’s Constitution of 1948 that declared health as a fundamental human right [1].

The role of the private sector in national health systems has been well acknowledged [1]. In the area of healthcare, the private sector comprises “all providers who exist outside the public sector, whether their aim is philanthropic or commercial, and whose aim is to treat illness or prevent disease” [3]. They could be classified into broad categories, such as for-profit and not-for-profit actors or formal and informal care providers [4] Formality is defined in terms of recognition of healthcare providers by a country’s regulatory and legal framework [4]. Unlike the formal private actors, informal providers (IPs) are not recognized by a country’s regulatory and legal framework even though they are recognized and sought by the clients they serve [5]. In addition to operating outside the purview of regulations, IPs usually have little or no officially recognized training [5]. Typically, they receive payments, in an undocumented fashion, from patients rather than institutions; and may be part of professional associations that do not have certification or regulatory authority [5].

IPs are broadly differentiated by the nature of their practice. They include drug sellers, traditional birth attendants (TBAs), untrained allopathic providers, traditional healers, faith healers and homeopaths. They also include people trained in one area, but practicing in another area (such as trained nurses consulting as physicians in their neighborhoods). Their services are utilized for a wide variety of interventions, including preventive, curative and restorative, and are sought out by care-seekers for three main reasons: proximity, ease of access, and familiarity to the community [5].

### The presence of informal providers in low-and-middle-income countries

IPs represent a significant portion of the healthcare delivery systems in low-and-middle-income countries (LMCs). They are the source of a large percentage of care delivered in South Asia and Sub-Saharan Africa [6]. For instance, they account for 55% of all providers in India [7], 77% in Uganda, and close to 96% in rural Bangladesh [5]. In the African Region, IPs account for 17% of primary care visits [2]. Access and utilization of their services for specific health conditions varies. In rural Mozambique for instance, about 43% of pregnant women deliver using TBAs [8] and in Uganda, 40% receive treatment for diarrhea from traditional healers [9]. For the service of drug vendors, it ranges from 35% to treat sexually transmitted infections in rural Uganda [9] to between 36 and 50% to treat common fever in rural Nigeria [10].

Some IPs also serve as complementary care providers, supporting formal healthcare systems in service delivery. In Zambia, trained TBAs provide counseling, referral and logistical support, including treatment adherent support, to formal care providers in resource-poor settings [11]. In rural Pakistan, TBAs assist community health midwives in normal deliveries, as well as referring high-risk cases to the formal health system [12]. Ghana has two complementary types of community health workers (CHWs) - formally and informally trained health service-supporting CHWs who support the formal healthcare system in rural and remote areas [13].

Although they are heavily utilized, IPs pose a great challenge to national health systems in LMCs [14]. Whereas the quality of care provided by the formal private providers is often perceived to be good and the industry may fill a gap in services that governments in LMCs might not afford to provide [1], the clinical services rendered by IPs are of substandard [15, 16]. Generally, IPs practice poor preventive medicine and do not adhere to national clinical guidelines [17]. They also have limited training across multiple health outcomes and lack the capacity to provide basic curative services [5]. For instance, TBAs with little or no training in modern techniques of reducing neonatal and maternal deaths assist women at childbirth in most LMCs [8]. This situation threatens the UHC objective of ensuring sufficient quality of healthcare for all. There is also the issue of regulating their activities. Governments in LMCs have weak governance and regulatory arrangements to effectively manage the activities of the informal sector. Countries with regulations find it difficult enforcing them because some IPs are located in remote and difficult areas to reach, while others are protected by community leaders who value their services. As such, most IPs engage in harmful, needless and wasteful medical practices, such as inadequate testing before diagnosis, dispensing of multiple drugs for a single episode, over-prescribing antibiotics and other medications, and carrying out unnecessary injections [5]. Many drug vendors, for instance, trade in dangerous drugs which are normally prescribed and supervised by qualified professionals, while others do not keep their drugs under proper storage conditions, thus rendering them ineffective [17]. There are also claims of “all purpose-efficacy” treatment of illnesses by traditional healers which have resulted in complications and more deaths among rural dwellers [18].

Despite these challenges, IPs play a critical role in healthcare delivery in LMCs. They fill significant gaps in formal healthcare provision, particularly, in countries with health worker shortages and where most qualified health professionals are largely concentrated in urban areas [19]. Also, unlike formal providers, IPs appear to be effectively reaching the hard-to-reach populations located in rural and remote areas. In such settings, they serve as the main care providers for poor populations without the means to travel to public facilities. Further, compared with both the public and the formal private providers, IPs are considered to have flexible working hours and offer more rapid services [19, 20]. Moreover, as much of their practice is contingent on the maintenance of good relationships with their communities, IPs tend to offer more flexible payment plans to their clients. As a result, they are able to buttress their community role and, thus, strengthen their business position [21].

IPs also have a significant influence on health belief systems in LMCs. Most often indigenous people believe that formally trained doctors are not fully equipped to address their health concerns, which have physical and spiritual dimensions [22]. Their beliefs and perceptions of ill-health are influenced by traditional healers who form an alternative health service in many rural communities. Many traditional African communities, for example, are of the view that ill-health is often caused by attacks from evil spirits. There is the belief that people with evil powers could cause other people they consider their enemies or disrespectful to become sick as a way of punishment. There is also the belief that disobeying taboos is another way people could become sick. These beliefs influence people’s behavior to use indigenous medical systems (informal) as an alternative healthcare service along with allopathic medicine (formal) [22].

### Informal providers and universal health coverage in low-and-middle-income countries

Considering the challenges and opportunities that IPs pose to health systems in LMCs, the key question is: should they be ignored or included in national efforts towards UHC? On the one hand, allowing them to pursue their own agenda means countries might not achieve the UHC objective of high-quality healthcare provision for all. On the other hand, leaving them behind might result in some LMCs’ inability to achieve the UHC objective of all people receiving needed health services of sufficient quantity, especially countries with no robust healthcare coverage. Considering the limited resources in most LMCs, ignoring entirely the role of IPs in national health systems might not be an option at this crucial moment. IPs who are not breaking any regulations could be harnessed to play an important role in achieving UHC in resource poor settings. Conversely, efforts should be made to limit those whose activities are considered harmful to population health.

The idea of formalizing IPs is hotly contested, but identifying those who are not breaking regulations and integrating them into the mainstream healthcare delivery system would not only ease the pressure on the overburdened system, but would also enable resource limited countries reach the poor and vulnerable populations to overcome inequalities in care delivery. For instance, evidence suggests that using institutional training, registration and licensing to make traditional medicine practitioners more reliable may decrease healthcare costs, as well as easing the increasing demand pressure on healthcare systems [23]. Tanzania’s Accredited Drug Dispensing Outlet (ADDO) program- where informal drug sellers have been integrated into the country’s mainstream healthcare system- is another demonstration that formalizing IPs is feasible and sustainable [24]. With appropriate training, TBAs could also play an important role in influencing the use of antenatal care services by either advising or referring pregnant women for registration or regular checkups.

Policy interventions to limit harmful informal healthcare practices could include enforcement of existing regulations and reducing the demand for informal services by improving the availability and performance of the formal healthcare sector. The frequent use of IPs reflects, in part, their widespread availability in rural and remote areas and the absence of trained medical professionals. The impact of IPs’ role “increases as the strength of the formal sector weakens” [5]. Thus, improving the availability and performance of the formal health sector might reduce the need for IPs. The use of community health committees could also help in improving the demand and supply of formal health services. Nigeria provides a classical example. In line with the country’s national health policy, each community is expected to have a community health committee, comprising the health worker in charge of the health facility to which the community is linked, representatives of traditional, voluntary, religious, women, youth and non-health occupational groups; and representatives of IPs in the community. These committee members provide information to reduce the market share controlled by IPs, as well as regulation to keep IPs at bay, while making the formal provider sector more competitive [25]. Another way to limit informal practices is through “universal health literacy”. Governments need to increase the level of health literacy in the general population to equip the general public to protect themselves against dangerous practices of IPs. Universal health literacy will also influence the health beliefs and health seeking behavior of indigenous populations in rural settings to utilize formal healthcare systems.

WHO has made progress towards recognizing and engaging the formal private health actors (both for-profit and not-for-profit) since the adoption of a resolution to engage the private sector in providing essential health services at the Sixty-third World Health Assembly (WHA) [2]. This engagement is seen in the WHO technical series on primary healthcare that has a separate focus on the role of the private sector [26]. The roll-out of the roadmap for public-private mix (PPM) for TB prevention and care [27] is another example of engaging the private sector in health service delivery. At the WHO regional level, 22 member states from the Eastern Mediterranean Region endorsed a framework for private sector engagement for UHC in 2018 [28]. The international body has assured member states of its continued support for UHC through private health sector service delivery governance. To this effect, an Advisory Group on private sector governance for UHC has been set up under the guidance of WHO’s Health System and Governance Department [29]. The Advisory Group has proposed a theory of change which envisions a health system that aligns the private service delivery actors to the public sector [2]. This alignment is driven by six governance behaviors: “building understanding” (providing reliable up-to-date information on current and future trends in health system performance), “fostering relations” (building and sustaining partnerships and coalitions), “enabling stakeholders” (ensuring that actors have the power to do their work), “aligning structures” (ensuring a fit between policy objectives and organizational structure and culture), “nurturing trust” (ensuring accountability), and “delivering strategy” (formulating strategic policy direction). Three types of private sector have been identified, under the proposed theory, for engagement: for-profit formal service delivery, not-for-profit service delivery, and for-profit informal service delivery [29]. Prioritizing the governance behaviors will depend on “the type of private sector that is dominant within a given country, the maturity of the country’s health system and the stage of growth of the private sector” [2]. This implies WHO does not support a “wholesale” integration of the private sector into the formal healthcare system. Thus, engaging the private sector, including IPs, should be country specific and the extent of engagement should be based on the strength of governance and regulatory arrangements in place.

## Conclusion

Increased interest in engaging the private health sector is a key opportunity for countries to leverage to meet the goals of UHC. IPs represent a significant portion of care providers in LMCs. However, while the business models of most IPs do not align well with UHC, governments do not have complete information about them, and lack the appropriate governance tools to help align their activities with national health systems and priorities [5]. Also, UHC is not only about the availability of healthcare services, but ensuring that these services are of sufficient quality. IPs may serve as informal extension of the formal healthcare delivery system in rural and resource-limited settings in LMCs. That notwithstanding, the services provided by some of these IPs are of sub-standard and harmful to people’s health. Thus, any policy decision to integrate this group of actors into the mainstream healthcare delivery system should be informed by research. Currently, there is a dearth of information on the types, size and utilization rates of the informal provider sector. There is also uncertainty surrounding the quality of service IPs provide, policies governing them and the overall impact they have within healthcare systems. Research tends to focus on the formal private sector, especially the non-profit providers [7]. Governments therefore need to sponsor comprehensive studies to document the basic characteristics of informal actors. The availability of such information would help in determining which types of IPs to engage and where in the scope of practice they could contribute positively towards the achievement of UHC.

## Data Availability

The data used in this analysis are available from the corresponding author on request.

## Abbreviations

CHWs: Community Health Workers
LMCs: Low-and-middle-income countries
IPs: Informal providers
SDGs: Sustainable Development Goals
UHC: Universal health coverage
WHA: World Health Assembly
WHO: World Health Organization
